# CpG-ODN Class C Mediated Immunostimulation in Rabbit Model of *Trypanosoma evansi* Infection

**DOI:** 10.1371/journal.pone.0127437

**Published:** 2015-06-03

**Authors:** Parveen Kumar, Rakesh Kumar, Balvinder Kumar Manuja, Harisankar Singha, Anshu Sharma, Nitin Virmani, Suresh Chandra Yadav, Anju Manuja

**Affiliations:** 1 National Research Centre on Equines, Sirsa road, Hisar-125001, Haryana, India; 2 Lala Lajpat Rai University of Veterinary and Animal Sciences, Hisar-125001, Haryana, India; Public Health Research Institute at RBHS, UNITED STATES

## Abstract

CpG oligodeoxynucleotides (CpG-ODN) stimulate immune cells from a wide spectrum of mammalian species. Class C CpG-ODN is relatively stable and has the combined immune effects of both A and B classes of CpG-ODN. *Trypanosoma evansi* produces the state of immuno-suppression in the infected hosts. The current chemotherapeutic agents against this parasite are limited in number and usually associated with severe side effects. The present work aimed to determine the immunostimulatory effects of CpG-ODN class C in *T*. *evansi* infected rabbits. Rabbits inoculated with CpG C and challenged with *T*. *evansi* resulted in delayed onset of clinical signs with reduced severity in comparison to that of *T*. *evansi* infected rabbits. The treatment also enhanced humoral immune responses. Histopathological findings in liver and spleen revealed enhancement of mononuclear cell infiltration and secondary B cell follicles. These results demonstrate that CpG-ODN class C, has immunostimulatory properties in rabbit model of trypanosomosis. The use of booster doses or sustained delivery of CpG-ODN will further elucidate the prolonged CpG-ODN generated immune responses.

## Introduction

Non-methylated cytosine-phosphate-guanosine (CpG) motifs present in viral and bacterial DNA are one of the pathogen associated molecular patterns (PAMPs) recognized by mammalian innate immune system [1]. These unmethylated CpG motifs (CpG-ODN) are recognized as a danger signal by the innate immune system of the vertebrates. Synthetic CpG oligodeoxynucleotides (CpG-ODN) mimic bacterial DNA and are shown to have potent immunostimulatory activity in vertebrates [2].

Three distinct classes of synthetic CpG-ODNs that differ in structure and function have been described [3,4].The class A ODN consists of phosphorothioate poly G sequences on both the 3’ and 5’ ends of a phosphodiester core containing CpG motifs [3]. This class of ODN is typically characterized by its ability to induce better *in vitro*natural killer (NK) activity and higher interferon-alpha (IFN-α) secretion by murine leukocytes than class B ODN [5,6]. The Class A ODNs are rapidly degraded *in vivo* with a half-life of nearly 5 min [7] and therefore, are rarely used for *in vivo* studies. In contrast, class B ODNs having a nuclease-resistant phosphorothioate backbone, are much more stable than class A ODNs and induce marked B cell proliferation *in vitro* but are poor at NK cell activation [8,9]. CpG-ODN class C contains a phosphorothioate backbone, and is relatively stable [10]. These ODNs have the combined immune effects of both classes A and B ODNs.

*Trypanosma evansi* is the causative agent of surra, one of the most common and widespread trypanosomal disease of domestic and wild animals. The parasite is transmitted mechanically by biting flies such as *Tabanus* and *Stomoxys* [11]. Though this trypanosome can infect most of the mammals, the horses and camelsare the principal hosts and represent the most significant sources of economic loss. Surra is endemic in Africa, Asia and South America, where many animals die during disease outbreaks each year. Trypanosomes are unusual among protozoan parasites with regard to their unique property of possessing the thick immunogenic surface coat which is known as variant surface glycoprotein (VSG) [12].These parasites modify their VSG constantly leading to antigenic variation and thus evade the immune system of the host [13].The resistance against this parasite was thought to be largely conferred by the adaptive immunity that comprises VSG-specific B and T lymphocyte responses [14]. The current chemotherapeutic agents are limited in number, usually associated with severe side effects and far from ideal. Antigenic variation, difficulties in large scale fly control, severe side effects of trypanocidal drugs, relapse of infection after treatment are the major hurdles in control of trypanosomosis.

Trypanosomes produce the state of immuno-suppression in the infected host which renders it more susceptible to secondary infections and results in poor immune response to bacterial and viral vaccines [15]. Therefore, considerable interest has been generated in finding ways to stimulate the innate immune system. Hemmi and his associates suggested the use of molecules that interact with pattern recognition receptors on immune cells to achieve this goal [16].

CpG-ODNs have been used to enhance innate immunity and for protection of experimental animal models from infections like *Listeria monocytogenes* [17] and *Francisella tularensis* [18]. CpG ODNs have also been shown to confer protection against *Trypanosoma cruzi* and *Trypanosoma brucei* infection [19–22] and malaria [23] in susceptible BALB/c mice. Considering the plethora of literature available on use of CpG-ODN as immuno-modulator and immuno-therapeutic, it was thought pertinent to study the efficacy of CpG-ODN to counter the immunosuppression caused by *T*. *evansi* infection. We have observed previously that co-culturing of *T*. *evansi* antigen in horse PBMC’s with CpG A and CpG C resulted in synergistic effect in eliciting the immune response [24].The present work aimed to determine the immuno-modulatory effects of CpG-ODN class C in rabbit model of *T*. *evansi*.

## Materials and Methods

### Animals and experimental design

This study was carried out in accordance with guidelines for the Care and Use of Laboratory Animals, issued by Committee for the Purpose of Control and Supervision of Experiments on Animals (CPCSEA), Government of India. The protocol was approved by Institutional Animal Ethics Committee of National Research Centre on Equines, Hisar, Haryana, India. Thirty healthy New Zealand white rabbits of either sex were procured from diseasefree small animal house. The age and weight of the rabbits ranged between 2.5–4 months and 1–2 kg, respectively.

Frozen stabilates of cryopreserved *T*. *evansi* maintained at Parasitology laboratory, NRCE, Hisar were expandedin mice for infection of rabbits. Briefly, Swiss albino mice were injected with 1x10^4^ trypanosomes intra-peritoneally (I/P). The peripheral blood from tail of mice was examined daily for scoring degree of parasitemia. Table 1 summarizes the treatments given to five groups of rabbits. First group of rabbits were injected intra-muscularly (I/M) with the mice blood in phosphate buffered saline with glucose (PBSG) containing 1×10^5^ trypanosomes on day 3 of experiment and kept as positive control. Synthetic ODNs containing unmethylated CpG-ODN 2395 (CpG C) having sequence 5’-tcgtcgttttcggcgcgcgccg-3’ (*Sigma Genosys*, India) was reconstituted using endotoxin free water. Two groups of rabbits (II and III) were given CpG C prepared in 10% oil-in-water emulsion (Sigma-Aldrich, St Louis, USA) I/M at the dose rate of 20 μg/kg body weight (b.wt). Second group of rabbits were challenged with 1×10^5^ trypanosomes on day 3 of experiment by injecting *T*. *evansi* mice blood I/M. The rabbits in group IV were injected with CpG-ODN class C alone at the dose rate of 20 μg/kg b.wt. I/M.The rabbits of group V (negative control) were injected with endotoxin-free PBS.

**Table 1:** Details of experimental groups and treatments.

Group	Experimental design
**I**	**Inoculated with 1x105 T. evansi (Horse strain) parasites/animal in phosphate buffered saline with glucose (PBSG) by i/m route on day 3 of experiment (Positive control).**
**II**	**Inoculated with CpG C (20**μ**g/kg body wt.) formulated with 10% oil-in-water emulsion on day 0 of experiment and challenged with 1x105 T. evansi (Horse strain) parasites/animal in PBSG by i/m route on day 3 of experiment.**
**III**	**Inoculated with CpG C (20**μ**g/kg body wt.) formulated with 10% oil-in-water emulsion on day 0 of experiment by i/m route.**
**IV**	**Inoculated with CpG C (20**μ**g/kg body wt.) dissolved in PBS on day 0 of experiment by i/m route.**
**V**	**Inoculated endotoxin-free PBS (100**μ**l/kg body wt.) on day 0 of experiment by i/m route (Negative control).**

### Clinical, haematological and biochemical observations

*In vivo* experiments performed, complied with the regulations set out by CPCSEA. Animals were monitored daily for seven weeks. Prolonged hyperthermia/hypothermia, (more than 72 hrs) and/or more than 15% pre-infection weight loss was considered as humane endpoints. The rectal temperature and clinical signs of all the animals were recorded daily. The change in the body weight of the rabbits of different groups was determined weekly. The blood samples were collected in tubes containing EDTA on days 0, 3, 7, 14, 21, 28, 35, 42 and 49. Plasma was separated by centrifugation and stored at -20°C until analysis. The blood haemoglobin was measured using Sahli’s acid haematin method. The blood glucose was measured on the spot using fresh drop of blood by blood glucometer. For demonstration of *T*. *evansi* parasites, wet smears were examined daily. The thin blood smears were also prepared, dried, stained with Giemsa stain and examined for detection of parasites and morphological changes in the blood cells.

### Estimation of immunoglobulin-G (IgG) concentration

The quantitative turbidimetric assay was used for the measurement of IgG in test samples [25]. The anti-rabbit IgG antibodies formed insoluble complexes when mixed with samples containing purified rabbit IgG (Invitrogen, Life technologies, Carlsbad, CA). The scattering of light by the immune-complexes to determine the IgG concentration in the sample was quantified by comparing with a calibrator of known IgG concentration (5 mg/ml to 0.625 mg/ml). The 50μl volume of each dilution was dispensed in duplicate in 96 well flat bottom microtitre plate (Greiner bio-one, Cellstar) and 50μl of anti-rabbit IgG prepared in tris buffer was added to these dilutions. Absorbance of each well was measured at 540 nm by ELISA plate reader (Biotek instruments, powerwave X2, USA) and plotted between absorbance and IgG concentration of each dilution. The IgG standard curve was used to determine the concentration of IgG in test samples.

### Estimation of *T*. *evansi* specific immunoglobulin-G

The whole cell lysate (WCL) *T*. *evansi* antigen was prepared using purified trypanosomes [26]. Parasite-specific antibody responses were measured by ELISA, as previously described with some modifications [27,28]. Briefly, 96-well plates were coated with 50 μl of 500 ng WCL *T*. *evansi* antigen in 0.1 M carbonate-bicarbonate buffer (pH 9.6) and incubated overnight at 4°C. After six washings with PBS containing 0.05% tween-20 (PBST), the wells were blocked with 5% skimmed milk in PBST for 1 h at 37°C. Subsequent to further six washings in PBST, 50 μl of 1:100 diluted plasma samples in blocking solution were added to the corresponding wells and incubated for 1 h at 37°C. After washing, equal quantity of 1:5000 dilution of goat anti-rabbit IgG-horseradish peroxidase conjugate (Sigma) was added to each well and incubated for 1 h at 37°C. After six washings and subsequent addition of 1:20 tetramethylbenzidene (TMB/H_2_O_2_) substrate solution, the reaction was stopped with 1 M sulphuric acid after 10-min incubation at room temperature. The absorbance was read at 450 nm by ELISA reader (Bio Tek, USA) and results were expressed as mean OD 450 of duplicate samples. The relative percent positivity (RPP) of the samples was determined using formula [(mean OD of test sample-mean OD of negative sample)/(mean OD of positive sample- mean OD of negative sample)]*100.

### Histopathological evaluation

The IgG levels were monitored for a period of seven weeks. At the end of the experiment, either animals were euthanized for histopathological studies or treated with trypanocidal drug quinapyramine sulfate. Animals were euthanized by injecting sodium thiopentone I/V.The procedure of euthanasia was quick and painless and in an atmosphere free from fear or anxiety. Liver and spleen of rabbits were dissected and fixed in 10% neutral buffered formalin. The tissues were washed thoroughly with water, processed by automatic tissue processor and then embedded in paraffin wax. The tissue pieces were sliced by microtome to about 3–4μm thickness. The slices were fixed on glass slides and stained with hematoxylin and eosin stain. All the stained slides were examined microscopically to observe pathological changes due to *T*. *evansi* infection and/or CpG treatment.

### Statistical analysis

Data were analyzed using the statistical software program *Sigmaplot* version 12.5. The differences in various clinical parameters within the groups and across the groups were investigated using one-way analysis of variance (ANOVA) applying non-parametric test. The means of the treatment groups were further compared using Holm-Sidak multiple comparison test. The data were analyzed statistically and p values <0.05 were considered significant.

## Results and Discussion

It is well known that CpG-ODNs activate innate immunity and has potential to protect animals against various infections [22]. Literature suggests that CpG ODN treatment reduced the severity of clinical signs of the diseases. CpG ODN treatment inrhesus macaques significantly reduced the severity of the lesions caused by a challenge with *Leishmania* infection [29]. We have observed synergistic effects of *T*. *evansi* antigen and CpGs co-culture on proliferation of horse PBMC [24]. In the present study, the rabbits were inoculated with CpG-ODN C with the aim to boost the immune response and then challenged with *T*. *evansi* to record the immuno-modulatory effects of CpG-ODN against *T*. *evansi*. Rabbits were taken as experimental model due to the chronic nature of the *T*. *evansi* infection in rabbits, mimicking naturally infected animals.

### Clinical signs

**Fig 1:**
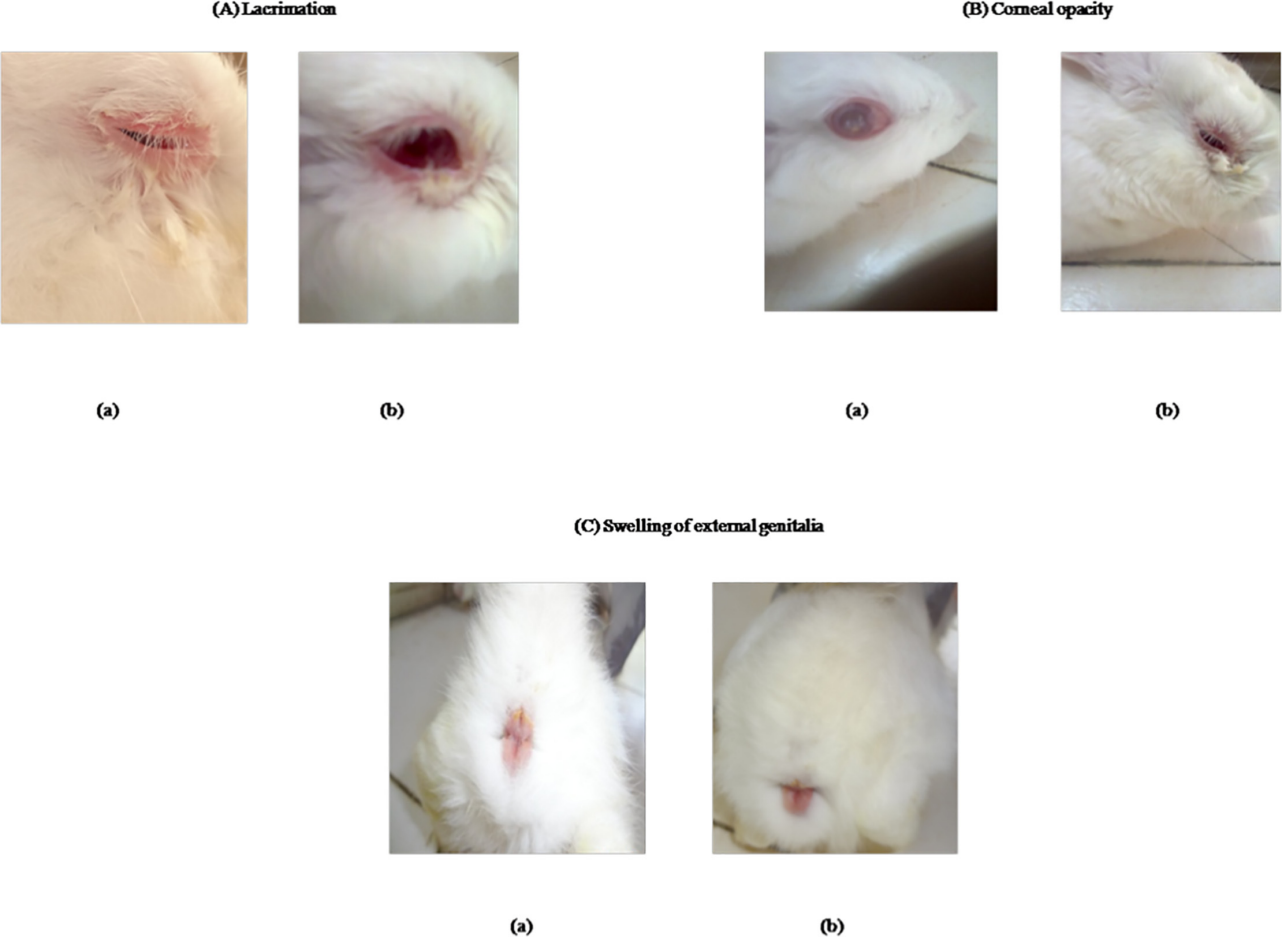
Photographs showing typical clinical signs in *Trypanosoma evansi* infection in rabbits. **A**. Lacrimation **B**. Corneal opacity **C**. Swelling of external genitalia. a. *T*. *evansi* infected rabbit b. CpG C treated rabbit challenged with *T*. *evansi*

Acute clinical signs *viz*. rise in rectal temperature, loss of appetite, drop in feed consumption and dullness appeared within days 3 to 5 post-infection in all rabbits of control group (Group I) whereas these signs appeared after day 5 post-infection in rabbits of CpG treated and *T*. *evansi* challenged group (Group II). Condition of none of the animal deteriorated to reach humane endpoint. Since the rabbits were in growing stage, we observed increase in the body weight in all the groups irrespective of *T*. *evansi* infection (Table A in S1 File). None of the rabbit showed hypothermia, prolonged hyperthermia (more than 72 hrs) or reduction in pre-infection body weight more than 15%. The clinical signs were rough hair coat, dullness, fluctuating rectal temperature, pale mucous membrane, swellings of external genitalia, incoordination of movement, lacrimation, deposition of white plaques in eyes and corneal opacity in some animals (Fig 1). Eyes were washed with 2% boric acid solution to provide relief to the rabbits from lacrimation.The clinical signs of *T*. *evansi* infection with reduced severity appeared within days 26 to 30 post-infection in positive control group of rabbits (group I) and within days 34 to 38 post-infection in CpG treated group (group II).Abdul-majeed *et al*. (2007) observed the clinical signs in rabbits including rise in temperature during the first three days after infection, loss of appetite, progressive emaciation, and refusal to walk due to recumbency, depression, conjunctivitis, corneal opacity, and anemia in most of the infected rabbits [30].

**Fig 2:**
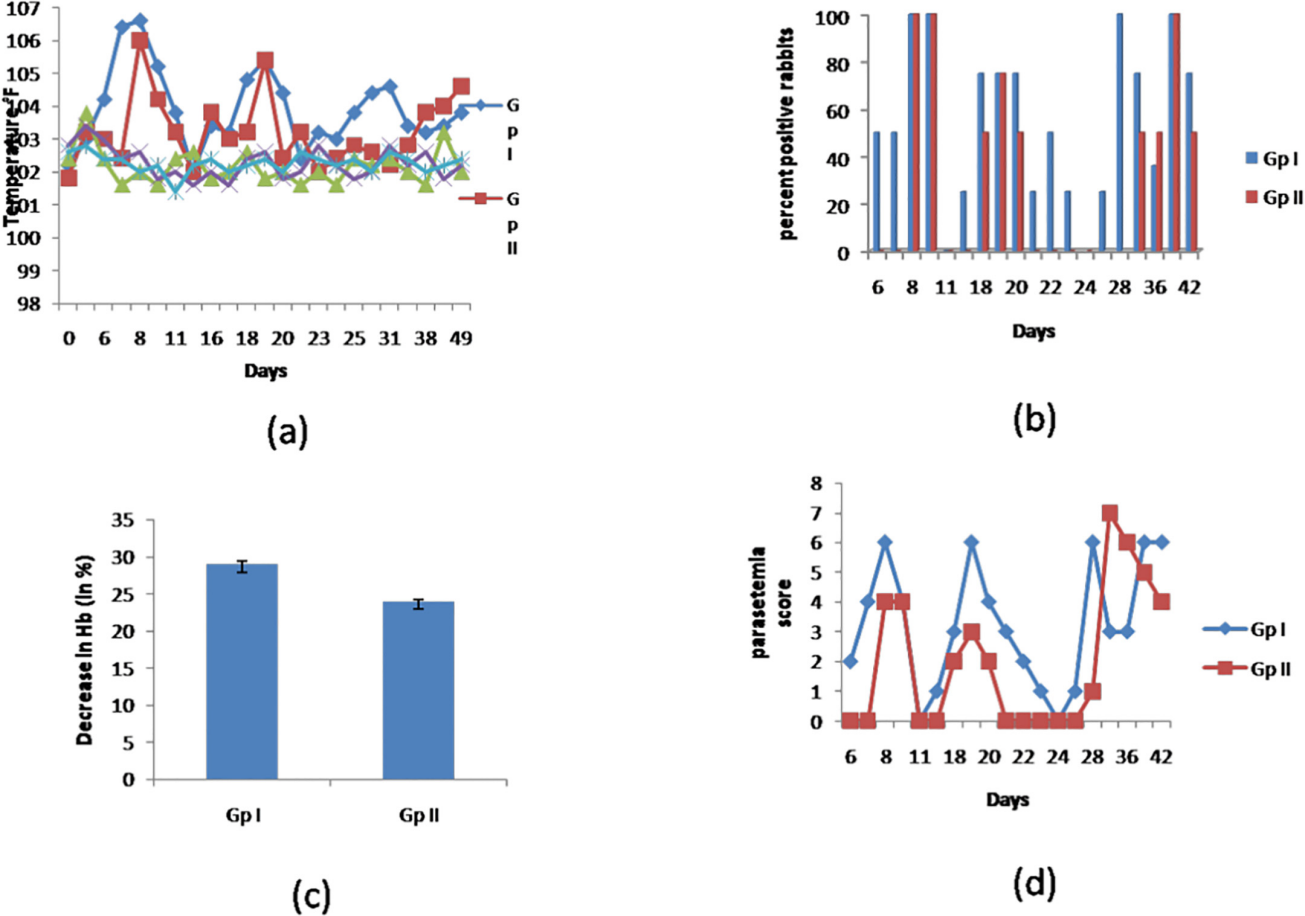
Effects of CpG-ODN C treatment in *Trypanosoma evansi* infected and uninfected rabbits. The rabbits of group I were infected with 1x10^5^ *T*. *evansi* parasites/animal. The group II rabbits were treated with CpG C formulated with 10% oil-in-water emulsion and then challenged with 1x10^5^ *T*. *evansi* parasites/animal. The rabbits of group III, IV and V were inoculated with formulated CpG C, CpG C alone and PBS as negative control, respectively. Effects of CpG-ODN treatment and/or *T*. *evansi* infection. **A**. Rectal temperature (°F). **B**. The number of rabbits showing parasitemia in wet blood film during the course of the disease in both the infected groups were determined and expressed in percentage. Figure B depicts the percent of rabbits of groups I and II showing parasitemia on different days. **C**. Parasitemic scores on different days in groups I and II. D. Percent decrease in haemoglobin level in groups infected with *T*. *evansi*.

Statistically significant changes in rectal temperature were reported in rabbits of two groups, which were experimentally infected with *T*. *evansi* (p<0.001). Slight increase in body temperature was observed on day 3 of experiment in all CpG treated groups. In positive control group, two animals reflected significant increase in rectal temperature on day 3 post-infection (PI) i.e. day 6 of experiment. On days 8 and 9, rectal temperatures in all the six animals were on peak (p<0.001). The second peak of temperature was reported at an interval of 8–11 days. Whereas, in CpG treated and *T*. *evansi* challenged rabbits (group II), duration and rise in temperature on the first two peaks was lower than that observed in the positive control group. The remarked delay in the third peak of fever was also observed. Effects of CpG-ODN treatment and/or *T*. *evansi* infection on rectal temperature (°F) of animals from each of five groups of rabbits is shown in Fig 2A. Clinical symptoms and other parameters of *Trypanosoma evansi* infected rabbits (Group I) and *Trypanosoma evansi* infected and challenged with CpG C rabbits (Group II) are summarized (Table B in S1 File).

### Haematological and parasitological observations

The parasites appeared in wet blood smears from rabbits of CpG treated and *T*. *evansi* challenged group (group II) on day 5 PI, second peak on day 15 to 17 and third peak appeared on day 28 PI and persisted till the end of experiment. The parasitemia was higher and its duration was longer during first two parasitemic peaks in *T*. *evansi* infected rabbits (group I) as compared to that of CpG treated and *T*. *evansi* challenged rabbits (group II),which is indicative of immunomodulatory effects of CpG-ODN inoculations, and coincided with higher IgG levels on day 7 of experiment in these rabbits. The fluctuating pyrexia coincided with inconsistent parasitemia. Recurrent episodes of parasitemia occur during the course of the disease (Table C in S1 File).The number of rabbits showing parasitemia in both the infected groups were determined and expressed in percent. Overall, 58.94% rabbits of positive control group, showed parasitemic peaks during 42 days in comparison to 36.71% of rabbits in group II. Fig 2B depicts the percent of rabbitsof groups I and II showing parasitemia on different days. Fig 2C shows the parasitemic scores shown by the rabbits of the two groups. The parasitemic scores were determined for each *T*. *evansi* infected group on different days, considering all the rabbits showing parasitemia in wet blood film (Table C in S1 File). The difference in the parasitemic scores between both the groups is statistically significant (p<0.012) for 28 days as determined by unpaired student’s-t test. After 28 days, the insignificant statistical difference in parasitemia suggests the need of booster dose of CpG. In a recent study in experimentally infected donkey mare, the parasite was not observed in wet blood film on microscopic examination, however in serum, a significant level of antitrypanosmal IgG antibodies in ELISA were present [31].

The mean haemoglobin level decreased from 12.63±0.45 g/dl to 8.97±0.29 g/dl in *T*. *evansi* infected rabbits (group I) and from 12.60±0.33 g/dl to 9.57±0.45 g/dl in CpG treated and *T*. *evansi* challenged rabbits (group II). The mean haemoglobin levels decreased by 28.9% in *T*. *evansi* infected rabbits (group I), whereas the significant decrease in Hb levels in CpG treated and *T*. *evansi* challenged rabbits (group II) is 23.9% (Fig 2D). The difference in the decrease of mean haemoglobin values between both the groups is statistically significant (p<0.0001) as determined by student’s-t test. The values decreased significantly from day 14 in both the groups I and II with respect to day 0 observations (Table 2). These decreased haemoglobin values were found to be statistically significant by one way ANOVA using pair wise multiple comparison (Holm-Sidak method). There was no significant alteration in haemoglobin values in group III, IV and V throughout the observation period of 49 days (Table 2).

**Table 2:** Effects of CpG ODN inoculation and/ or *Trypanosoma evansi* infection on mean haemoglobin values (g/dl) in different groups of rabbits.

Groups/Days	0	3	7	14	21	28	35	42	49
**I**	12.63^a^ ^c^ ^e^ ^h^	12.67^a^ ^c^ ^e^ ^h^	11.97^a^ ^c^ ^e^ ^h^	11.05^a^ ^c^ ^e^ *	10.32 ^a^ ^c^ *	9.83^h^ ^r^ *	9.4^e^ ^p^ *	9.1^c^ ^n^ *	8.97^a^ ^k^ *
	(0.45)	(0.34)	(0.53)	(0.43)	(0.48)	(0.53)	(0.6)	(0.36)	(0.29)
**II**	12.6 ^b^ ^d^ ^f^	12.63 ^b^ ^d^ ^f^	11.9 ^b^ ^d^ ^f^	11.3 ^b^ ^d^ ^f^ *	10.73^b^ ^d^ *	10.47*	9.93^f^ ^q^ *	9.6^d^ ^o^ *	9.57^b^ ^m^ *
	(0.33)	(0.39)	(0.43)	(0.47)	(0.5)	(0.75)	(0.56)	(0.52)	(0.45)
**III**	12.32	11.92	11.84	12.12	12.2	12.32 ^r^	12.36 ^p^ ^q^	12.44 ^n^ ^o^	12.48 ^k^ ^m^
	(0.68)	(0.66)	(0.56)	(0.77)	(0.57)	(0.48)	(0.6)	(0.5)	(0.37)
**IV**	12.4	12.56	12.48	12.48	12.52	12.8 ^r^	12.72 ^p^ ^q^	12.56 ^n^ ^o^	12.68 ^k^ ^m^
	(0.59)	(0.46)	(0.37)	(0.35)	(0.27)	(0.28)	(0.2)	(0.23)	(0.27)
**V**	12.47	12.37	12.33	12.57	12.6	12.87^r^	12.87 ^p^ ^q^	12.67 ^n^ ^o^	12.37 ^k^ ^m^
	(0.41)	(0.31)	(0.46)	(0.29)	(0.33)	(0.25)	(0.25)	(0.25)	(0.27)

* Significant decrease in values with respect to day 0 within groups (p<0.05)

^a c e h b d f.^ The values are significantly different within group across the days (p<0.050)

^p q r k m n o.^ The values are significantly different across the groups (p<0.050).

### Biochemical studies

The mean values of blood glucose (g/dl) along with their respective standard deviations of different groups of rabbits are shown in Table 3.The significant fall in mean blood glucose values was reported on days 7 and 35 in positive control group (group I) at p<0.001, whereas mean blood glucose values did not show any significant alteration in CpG treated and *T*. *evansi* challenged (group II) rabbits (p<0.05).

**Table 3:** Effects of CpG ODN inoculation and/ or *Trypanosoma evansi* infection on mean blood glucose values (g/dl) in different groups of rabbits.

Groups/Days	0	3	7	14	21	28	35	42
**I**	129.67 ^**a**^	131.33 ^**a**^	93^**a**^ ^,^ ^**b**^ *	106.5	105.5	104.17	98.5 ^**a**^ *	111
	(5.88)	(6.18)	(8.91)	(16.14)	(27.82)	(19.85)	(8.66)	(10.50)
**II**	124.8	126.6	106.4	115.2	116.2	109.4	118.6	115.8
	(6.52)	(5.43)	(7)	(7.96)	(15.16)	(11.11)	(11.48)	(9.66)
**III**	125	125	137.5 ^**b**^	129	126.5	121	130	120.5
	(8)	(4)	(7.5)	(1)	(5.5)	(3)	(1)	(2.5)
**IV**	129.67	125.33	131.67 ^**b**^	132.33	131	130	129.33	125.67
	(2.49)	(8.26)	(2.05)	(4.03)	(5.72)	(1.63)	(7.59)	(3.68)
**V**	123.5	122	124.5	137.5	134.5	124	131.5	120.5
	(2.5)	(2)	(7.5)	(0.5)	(0.5)	(2)	(3.5)	(1.5)

*Means with superscripts vary significantly with respect to days 0 and 3 (p<0.05)

a. The values are significantly different within group across the days (p<0.050)

b. The values are significantly different across the groups (p<0.050).

Hypoglycemia in *T*. *evansi* infected animals has already been reported [32]. It might be due to the high metabolic rate caused by fever, hepatocyte degeneration or glucose consumption by the trypanosomes [33].

### Immunoglobulin-G concentration

**Fig 3:**
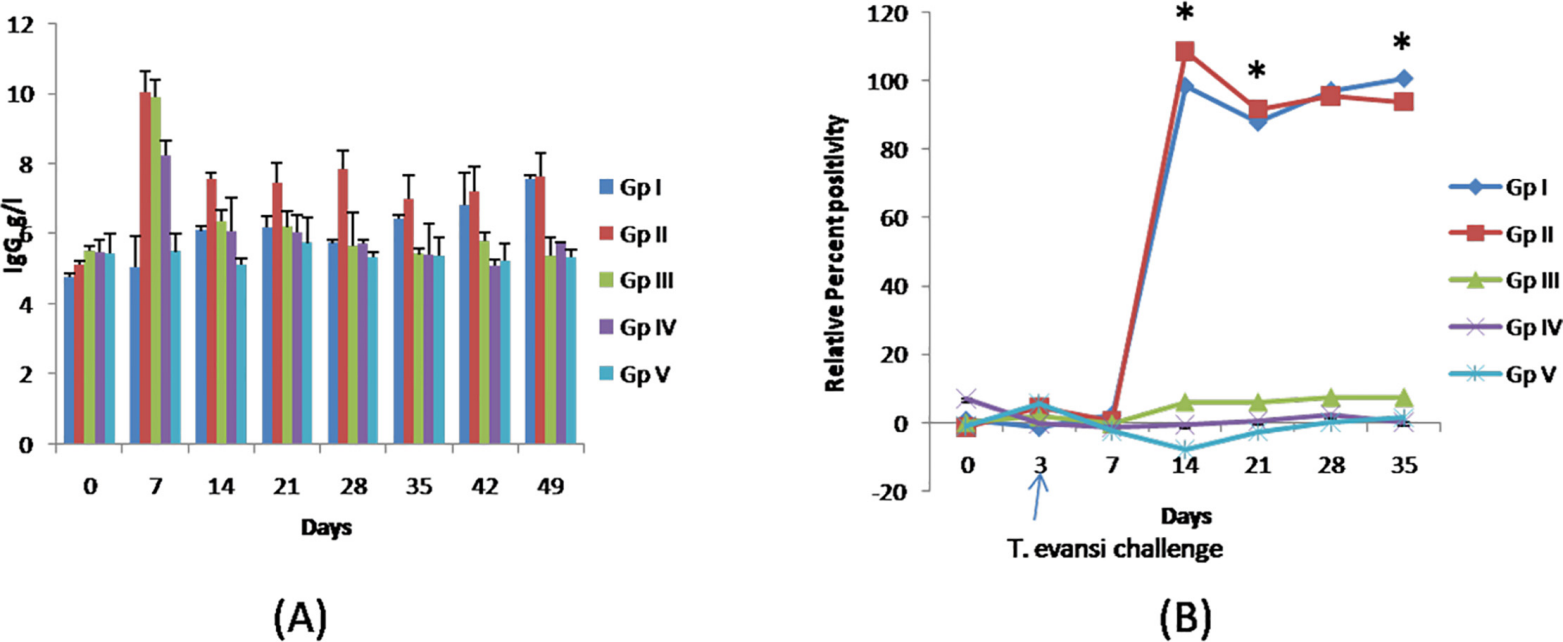
Effects of CpG ODN inoculation and/ or *T*. *evansi* infection. **A**. Immunoglobulin G values (g/l) in different groups of rabbits. The anti-rabbit IgG antibodies formed insoluble complexes when mixed with samples containing purified rabbit IgG. IgG concentration in the sample was quantified by comparing with a calibrator of known IgG concentration. **B**. Relative percent positivity for *T*. *evansi* specific Immunoglobulin G. *T*. *evansi* specific IgG levels were determined by ELISA and expressed as relative percent positivity with respect to known *T*. *evansi*positive serum sample.

The IgG levels were the highest on day 7 of experiment in groups II, III and IV i.e. groups treated with formulated CpGs (group II and III) and CpGs alone (group IV) (Fig 3A). Furthermore, the levels of IgG were higher in groups II and III than group IV. The values of IgG decreased in all these three groups during next observations and decrease in IgG values of group II was lesser in comparison to that of group III and IV. On day 35 onwards the regular elevation in the IgG values of group I was observed.

The highest IgG concentration was observed after CpG inoculation in (*T*. *evansi* infected and uninfected) rabbits treated with formulated CpGs and CpGs alone. CpG-ODN provokes humoral immune responses with less toxicity, superior to those induced by alum [34,35]. The values of IgG in rabbits receiving CpG-ODN decreased later which might be indicative of degradation of CpG-ODN, suggesting requirement of booster doses. After day 35 of experiment the regular elevation in the IgG values of *T*. *evansi* infected rabbits was observed which supported the earlier observation of increased plasma globulin levels in *T*. *evansi* infected donkeys [36]. Similar observations have been documented in other mammalian hosts infected with *T*. *evansi* [37, 38].

### *Trypanosoma evansi* specific immunoglobulin G

The detectable antibody levels against *T*. *evansi* were observed 11th dpi (or day 14 post CpG treatment) onwards in all the *T*. *evansi* infected rabbits in both the groups (Fig 3B). The plasma samples of five rabbits showed increasing trend of antibody titres from day 11 (98.49%) onwards and peak antibody titres were observed on 35^th^ dpi in Group I (positive control). In Group II peak antibody titres were observed on day 11 dpi (108.78%). The levels of *T*. *evansi* specific IgG levels were significantly higher than the rabbits in group I on day’s upto 21 days post CpG treatment or 18 dpi, which might be due to degradation of CpG-ODN. The regular elevation in the IgG values of *T*. *evansi* infected rabbits was because of cumulative effect and increasing number of blood parasites. Thereafter, antibody titres decreased as compared to rabbits of Group I. Similar observations for both the groups of rabbits were observed in IgG concentrations measured by quantitative turbidity test. Determination of *T*. *evansi* specific IgG levels can be compared between two groups only, which are receiving *T*. *evansi* parasite, but estimation of IgG levels using quantitative turbidity test provide comparison amongst all the groups irrespective of *T*. *evansi* infection.

### Histopathological evaluation

**Fig 4:**
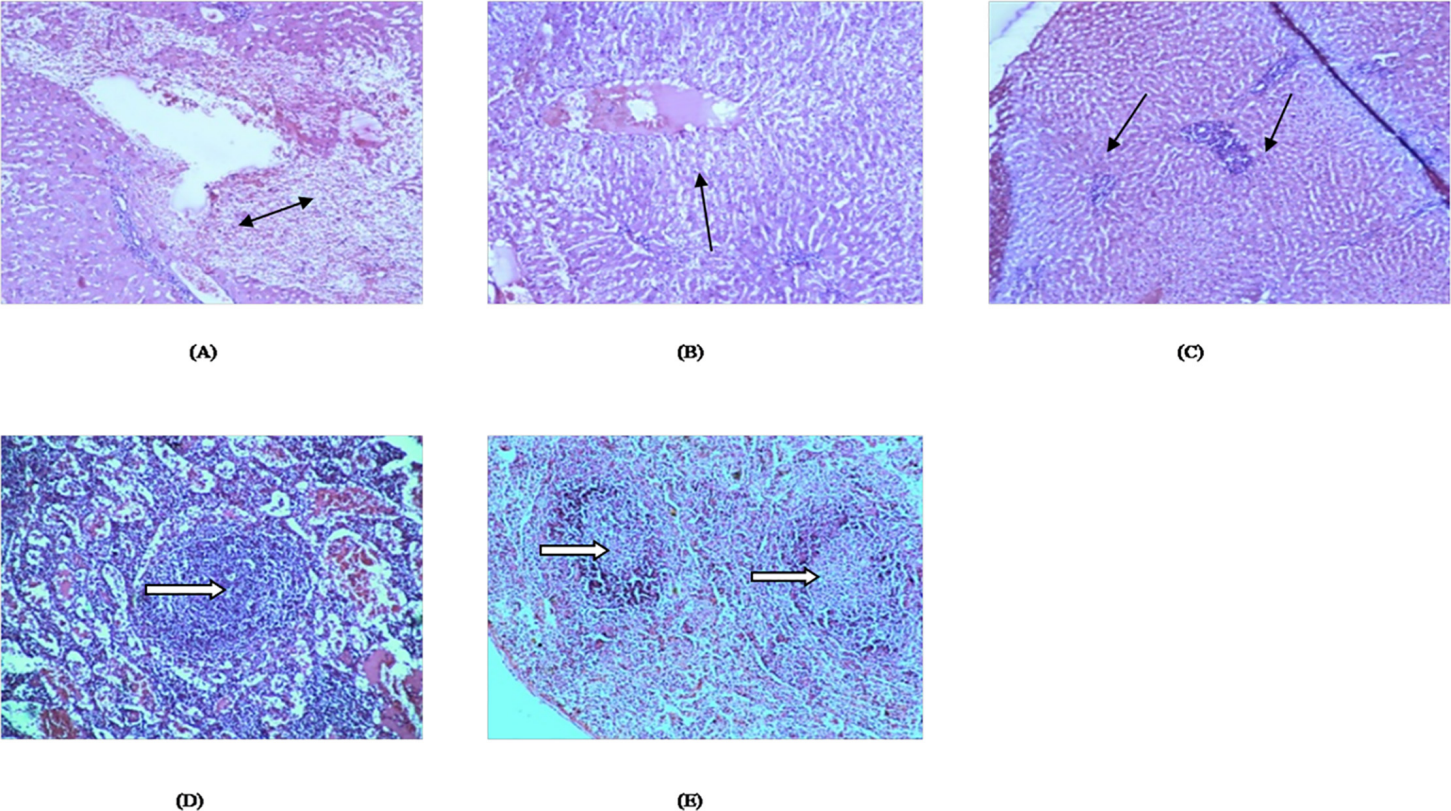
Histopathological changes in liver and spleen of *Trypanosoma evansi* infected and uninfected rabbits in response to CpG-ODN treatment. **A**. Liver tissue of *T*. *evansi* infected (positive control) group I, extensive necrosis and loss of normal hepatic architecture is shown by double arrow (H&E 400X)**. B**. Liver tissue of group II (CpG C treated and challenged with *T*. *evansi*), decreased severity of necrosis (black colored arrow) (H&E 400X). **C**. Liver tissue of group III (treated with formulated CpG alone),mononuclear cell infiltration in portal triad (black colored arrow) (H&E 400X). **D**. Spleen *T*. *evansi* infected (positive control) group I, secondary follicles (white block arrows), haemorrhages and edematous fluid(H&E 100X)**. E**. Spleen of group II CpG C treated and challenged with *T*. *evansi*. Secondary follicles comparatively more in number (white block arrows) (H&E 100X)

At necropsy, rabbits of positive control group (group I) and rabbits of CpG treated and *T*. *evansi* challenged group (group II) showed splenomegaly and multifocal areas of necrosis in spleen. Similar observations were reported in *T*. *evansi* infected donkeys and rabbits [36,39]. Histopathological changes in liver of *T*. *evansi* infected (group I) rabbits revealed hydropic degeneration of hepatocytes, progressive destruction of hepatic parenchyma, dilated sinusoids, heamorrhages in hepatic parenchyma and the inflammatory reaction extended from the portal tract to the parenchyma causing extensive hepatic necrosis and loss of normal hepatic architecture (Fig 4A). Hepatomegaly, congestion, necrotic foci and destruction of hepatocytes with infiltration of inflammatory cells were observed in the liver of *T*. *evansi* infected rats, buffaloes and goats also [40–43]. In rabbits treated with CpG C and challenged with *T*. *evansi* (group II), there was mild to moderate hydropic degeneration, mononuclear cell infiltrations and decreased severity of necrosis (Fig 4B) whereas in group III (treated with formulated CpG), the areas of mononuclear cell infiltration in portal triad along with mild hydropic degeneration of hepatocytes were observed (Fig 4C). Histopathological changes in spleen of *T*. *evansi* infected (group I) rabbits revealed formation of secondary follicles, moderate lymphocytic necrosis, haemorrhages and edematous fluid (Fig 4D) whereas in spleen of group II (CpG C treated and challenged with *T*. *evansi*) secondary follicles were comparatively more in number as compared to group I rabbits (Fig 4E). Depletion of lymphocytes and necrosis was also observed. Diseases caused by trypanosomes induce the formation of high levels of systemic antigen-antibody immune complexes and their consequent deposition in the heart, liver, brain and kidneys may possibly play a role in tissue damage [44]. However, some reports indicated that trypanosomes can cause tissue inflammation directly as a result of the infection [42,45]. The observed spleen lesions with follicular hyperplasia might be indicative of an immunological response by the infected rabbits. Following CpG-ODN stimulations, splenomegaly accompanied by proliferation of splenic B cells was also reported in mice [46].

This study was performed to observe the impact of single dose of CpG-ODN class C on *T*. *evansi* infected animals. The impact of booster doses of CpG-ODN can also be explored to prolong the CpG-ODN generated immune responses. Recently, multifunctional magnetic nanoparticles loaded with CpG- ODNs were used to show the impact of immune activation on human head and neck squamous cell carcinoma cells [47,48]. Earlier, we reported that the nano-delivery of quinapyramine sulfate-loaded nanoparticles provided sustained release of quinapyramine sulfate and is highly effective in *T*. *evansi* infected mice [49]. Thus, either nano delivery of CpGs in form of sustained release formulations or booster doses can be tried in this context.

## Conclusion

We observed delayed onset of clinical signs with reduced severity in CpG treated and *T*. *evansi* challenged rabbitsas compared to *T*. *evansi*infectedrabbits. It also enhanced humoral immune responses. Furthermore, histopathological findings in liver and spleen revealed that CpG-ODN induced enhancement of mononuclear cell infiltration and secondary B cell follicles. The reduction in severity of tissue damage in CpG treated and *T*. *evansi* challenged rabbits may beattributedto the high IgG values in this group of rabbits. It has been demonstrated that CpG-ODN class C, have immunostimulatory properties against *T*. *evansi* in rabbit model for trypanosomosis. The use of booster doses or sustained delivery of CpG-ODN will further elucidate the prolonged CpG-ODN generated immune responses.

## Future Perspective

In endemic areas, the disease increases significantly during the rainy season due to highbiting fly populations. The property of trypanosomes to rapidly change their surface glycoproteins to avoid the immune is the major obstacle to provide any vaccine against the parasite. Approaches to enhance the innate immune response in the animals either by CpG-ODN can be exploited in endemic areas with low infection or with the antitrypanosomal treatment to avoid relapse of infection.

The combined CpG ODN C along with conventional chemotherapy against *T*. *evansi* may provide new alternatives to control the disease with reduced frequency and doses of the treatment with trypanocidal drugs. Novel approaches for trypanocidal therapy along with these potential new molecules to enhance their therapeutic value need further elucidation.

## Supporting Information

S1 File Supporting information. Table A. Effects of CpG ODN inoculation and/ or *Trypanosoma evansi* infection on body weight (percent increase) in different groups of rabbits. Table B. Clinical symptoms, hematological and biochemistry parameters of *Trypanosoma evansi* infected rabbits (Group I) and *Trypanosoma evansi* infected and challenged rabbits with CpG C (Group II). Table C. Parasitemia shown by *Trypanosoma evansi* infected rabbits (Group I) and *Trypanosoma evansi* infected and challenged with CpG C rabbits (Group II). *T*. *evansi* Infection was given on day 3.
(DOCX)
