# Supplementary material for: CpG-ODN Class C Mediated Immunostimulation in Rabbit Model of Trypanosoma evansi Infection
Source: PLoS One. 2015 Jun 3;10(6):e0127437. doi: 10.1371/journal.pone.0127437 (PMC4454682; doi:10.1371/journal.pone.0127437)
Supplement: S1 File — Table A. Effects of CpG ODN inoculation and/ or Trypanosoma evansi infection on body weight (percent increase) in different groups of rabbits. Table B. Clinical symptoms, hematological and biochemistry parameters of Trypanosoma evansi infected rabbits (Group I) and Trypanosoma evansi infected and challenged rabbits with CpG C (Group II). Table C. Parasitemia shown by Trypanosoma evansi infected rabbits (Group I) and Trypanosoma evansi infected and challenged with CpG C rabbits (Group II). T. evansi Infection was given on day 3. (DOCX) [file pone.0127437.s001.docx]

**Table A.** Effects of CpG ODN inoculation and/ or *Trypanosoma evansi* infection on body weight (percent increase) in different groups of rabbits**.**

| **Days**  **Gps** | **3** | **7** | **14** | **21** | **28** | **35** | **42** | **49** |
| --- | --- | --- | --- | --- | --- | --- | --- | --- |
| I | 0 | -4.73  (3.14) | -4.13  (4.48) | -7.26  (2.52) | -0.85  (1.62) | 8.81  (2.81) | 8.76  (9.36) | 4.30  (2.52) |
| II | 0 | -2.29  (1.26) | 4.22  (2.69) | -0.76  (1.93) | 4.10  (7.09) | 5.53  (3.47) | 8.64  (5.17) | 5.93  (4.93) |
| III | 0 | -0.05  (0.46) | 1.19  (1.76) | 3.69  (1.65) | 13.65  (1.39) | 18.94  (0.20) | 17.25  (0.00) | 14.17  (2.13) |
| IV | 0 | -0.25  (1.38) | -0.26  (2.95) | 0.80  (2.70) | 9.61  (3.64) | 14.57  (8.45) | 16.38  (7.10) | 16.66  (5.28) |
| V | 0 | -1.78  (3.06) | 0.00  (2.30) | 2.93  (9.75) | 19.10  (4.83) | 22.17  (2.33) | 23.69  (0.73) | 22.80  (1.17) |

Standard deviations of respective means are given in parenthesis. *Trypanosoma evansi* infection was given on day 3.

**Table B.** Clinical symptoms, hematological and biochemistry parameters of *Trypanosoma evansi* infected rabbits (Group I) and *Trypanosoma evansi* infected and challenged rabbits with CpG C (Group II)

|  | Group I  (*Trypanosoma evansi* infected) | Group II  (*Trypanosoma evansi* infected and challenged with CpG C) |
| --- | --- | --- |
| Initial rise in rectal temperature | High rise of temperature 3-5 PI | Slight rise Day 3 after CpG  High rise in temperature after day 7 |
| Temperature peaks | Day 3-6 PI  Day 11-14 PI  Day 26-28 | Delay in peaks with reduced duration and rise in temperature |
| Swellings of external genitalia | Days 27-30 | Day 34-36 |
| Lacrimation | Days 27-30 | Day 34-36 |
| Deposition of white plaques in eyes | Days 27-30 | Day 34-36 |
| Corneal opacity | Days 27-30 | Day 34-36 |
| Decrease of mean haemoglobin values | 28.9 % | 23.9 % |
| Blood glucose | Significant fall on day 7 and day 35 | No significant alteration |
| Pre-infection body weight loss/gain (%) | Day 7 (-3.98%)  Day 14 (-0.67%)  Day 21 (-3.26%) | Day 7 (-1.86)  Day 14 (3.12)  Day 21 (-2) |

**Table C.** Parasitemia shown by *Trypanosoma evansi* infected rabbits (Group I) and *Trypanosoma evansi* infected and challenged with CpG C rabbits (Group II*). T. evansi* Infection was given on day 3

| Group/ animal no | I(1) | I (2) | I(3) | I(4) | II(1) | II(2) | II(3) | II(4) |
| --- | --- | --- | --- | --- | --- | --- | --- | --- |
| Days |  |  |  |  |  |  |  |  |
| 6 | **-** | **-** | **+** | **+** | **-** | **-** | **-** | **-** |
| 7 | **-** | **-** | **++** | **++** | **-** | **-** | **-** | **-** |
| 8 | **+** | **+** | **++** | **++** | **+** | **+** | **+** | **+** |
| 9 | **+** | **+** | **+** | **+** | **+** | **+** | **+** | **+** |
| 11 | **-** | **-** | **-** | **-** | **-** | **-** | **-** | **-** |
| 17 | **-** | **-** | **-** | **+** | **-** |  | **-** | **-** |
| 18 | **+** | **-** | **+** | **+** | **+** | **+** | **-** | **-** |
| 19 | **++** | **-** | **++** | **++** | **+** | **+** | **-** | **+** |
| 20 | **+** | **-** | **+** | **++** | **-** | **-** | **+** | **+** |
| 21 | **-** | **-** | **-** | **+++** | **-** | **-** | **-** | **-** |
| 22 | **-** | **+** | **-** | **+** | **-** | **-** | **-** | **-** |
| 23 |  | + | - | - | - | - | - | - |
| 24 | - | - | - | - | - | - | - | - |
| 27 | - | - | + | - | - | - | - | - |
| 28 | + | + | ++ | ++ | + |  |  |  |
| 31 | - | ++ | +/- | +/- | - | - | ++++ | +++ |
| 36 | - | + | + | +/- | ++++ | - | + | + |
| 39 | +++ | ++ | +/- | +/- | ++ | ++ | +/- | + |
| 42 | ++ | ++ | ++ | - | - | ++ | - | ++ |
